# Primary school students’ mental health in Uganda and its association with school violence, connectedness, and school characteristics: a cross-sectional study

**DOI:** 10.1186/s12889-016-3351-z

**Published:** 2016-07-29

**Authors:** Barbara F. Thumann, Ula Nur, Dipak Naker, Karen M. Devries

**Affiliations:** 1 Department of Epidemiological Methods and Etiological Research, Leibniz Institute for Prevention Research and Epidemiology – BIPS, Achterstrasse 30, 28359 Bremen, Germany; 2 Department of Non-Communicable Disease Epidemiology, London School of Hygiene and Tropical Medicine, Keppel Street, London, WC1E 7HT UK; 3 Raising Voices, 16 Tufnell Drive, Kamwokya, P. O. Box 6770, Kampala, Uganda; 4 Department of Global Health and Development, London School of Hygiene and Tropical Medicine, 15-17 Tavistock Place, London, WC1H 9SH UK

**Keywords:** Children, Adolescents, Mental health, Violence, School, Uganda

## Abstract

**Background:**

Few studies have explored risk factors for poor mental health in Ugandan primary schools. This study investigated whether individual- and contextual-level school-related factors including violence from school staff and other students, connectedness to school and peers, as well as school size and urban/rural location, were associated with mental health difficulties in Ugandan children. We also examined whether associations between violence exposure at school and mental health were mediated by connectedness as well as whether associations were different for boys and girls.

**Methods:**

The analytic sample consisted of 3,565 students from 42 primary schools participating in the Good Schools Study. Data were collected through individual interviews conducted in June and July 2012. Mental health was measured using the Strengths and Difficulties Questionnaire. Multilevel logistic regression was applied to investigate factors associated with mental health difficulties.

**Results:**

Experiences of violence from school staff and other students in the past week were strongly associated with mental health difficulties (OR = 1.58, 95 % CI 1.31 to 1.90 and 1.81, 1.47 to 2.23, respectively). Children with a low school connectedness had 1.43 times (1.11 to 1.83) the odds of mental health difficulties compared to those with a high school connectedness. The OR comparing children never feeling close to other students at their school with those always feeling close was 1.86 (1.18 to 2.93). The effect of violence on mental health was not mediated through the connectedness variables. School size was not related to mental health difficulties, but attending an urban school increased the odds of mental health difficulties after accounting for other factors. We did not find evidence that the effect of one or more of the exposures on the outcome differed between boys and girls.

**Conclusions:**

These findings suggest that violence in school and low connectedness to school and peers are independently associated with mental health difficulties and interventions should address both concurrently. Extra support may be needed for students in urban schools.

**Trial registration:**

Clinicaltrials.gov NCT01678846. Registered 24 August 2012.

## Background

Mental health problems occur in 10 to 20 % of children worldwide [1]. It is known that mental health problems in childhood tend to persist into adulthood [2] and apart from the suffering they cause for every affected individual, mental illness also entails a large economic burden through the depletion of labour and the resources needed for care [3]. Early prevention is regarded as a sustainable and probably cost-effective method to reduce the burden of mental disorders - especially in low-income countries where resources for care for mental disorders are low [4, 5].

Schools are ideal places for prevention and health promotion because interventions can be conducted conveniently in this setting and reach a high number of children and adolescents [6]. Furthermore, the school itself is a place where children’s mental health can be strengthened [2], for example by increasing school connectedness. School connectedness has been defined as the belief by students that adults in the school care about their learning as well as about them as individuals [7]. Studies conducted in high-income countries have highlighted school connectedness as a particularly important protective factor for a range of adverse mental health outcomes in youth [8–11]. In addition, student participation might be beneficial for mental health of students, although existing research is rather sparse and contradictory [12, 13].

However, school can also be a place where children’s mental health is harmed, for example through experiences of violence. In Uganda, for example, corporal punishment still exists despite an official ban in 1997 by the Ministry of Education and Sports. With regard to violence from peers, bullying, as one type of school violence [14], is a well-known risk factor for poor mental health of students in high-income [15–18] as well as in low-income countries [19, 20].

In general, most research on the association between school-related factors and mental health of students has been conducted in high-income countries. However, both the school environment and the epidemiology of mental disorders in high-income countries differ substantially from settings like Uganda.

In Uganda, violence from peers is common [21], as well as violence from school staff [22], and violence from school staff is associated with poor mental health [22]. However, it is not clear what the mechanism for this relationship is. It is possible that this relationship is in fact mediated by poor connectedness to school and other students (peers) but we identified no studies testing this pathway. Similarly, student participation might impact on school connectedness and in turn influence students’ mental health. It is also not known whether contextual-level variables, such as being in a larger school and being in a rural or urban school, impact on students’ likelihood of having mental health difficulties in low income settings. Understanding these relationships will give us valuable insight into how to design effective interventions to improve student mental health outside high -income countries.

**Fig. 1:**
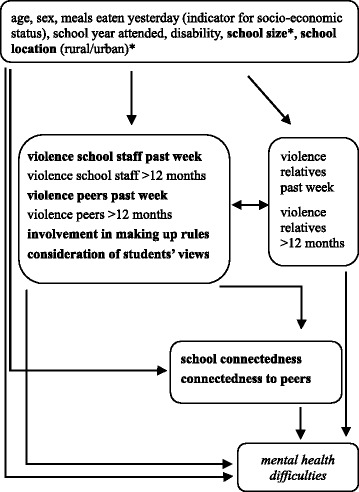
Conceptual framework of the hypothesized associations between the school-related factors of interest (in bold) and mental health difficulties (in italics) of students under consideration of various confounders (unformatted). Variables measured at school-level are denoted with an asterisk (*)

In this study we analyze data gathered from students in Uganda. Our aims were to (1) examine the strength of association between school-related factors (violence from school staff and peers, participation, connectedness, school size, school location) and mental health of students, (2) assess whether connectedness mediates the associations between violence and mental health and participation and mental health, respectively, and (3) evaluate whether the effect of one or more of the individual-level exposures of interest on the outcome differs between boys and girls (effect modification). We hypothesized that mental health difficulties are associated with experiences of violence, low participation in school life, low connectedness to school and peers, large school size and urban location of the school (Fig. 1).

## Methods

### Participants

This study used baseline data from the Good Schools Study, a cluster randomized controlled trial conducted in Luwero District in Uganda (registered at clinicaltrials.gov, NCT01678846) [23, 24].

The participants were selected in two stages. Firstly, after excluding 97 very small schools with less than 40 registered students in year 5 (Primary 5) and 20 schools with existing governance interventions, 42 schools were randomly selected from a list of the remaining 151 schools in the district. This sample represents 80 % of primary school students in the district. Secondly, a maximum of 130 students per school were selected from lists of all Primary 5, 6, and 7 students by simple random sampling.

### Procedure

Parents were informed about the study and the possibility to opt their child out from participating [23]. Informed written consent was sought from each selected student. The data were collected during individual interviews conducted in June and July 2012. Students were notified that there were no right or wrong answers and that all information would be kept private. Students disclosing serious maltreatment were referred to local child protection services [25]. Ethical approval for the study was obtained from the Ugandan National Council for Science and Technology (SS 2520) and the Ethics Committee of the London School of Hygiene and Tropical Medicine (#6183). The study protocol was published in detail elsewhere [23].

### Instruments

The following measures from the interview were used for this study. All measures were adapted and cognitively tested for understanding via an iterative process with a small group of primary school children and then pilot tested with 697 children attending Primary 5, 6, and 7 to explore item distributions before the main survey. The exact wording of the questions used for the creation of the individual-level exposure variables is displayed in Table 1.

**Table 1:** Description of individual-level exposure variables

Variable name	Items	Coding
Violence school staff past week	Emotional violence	Coded 1 if answered yes about past week experience to any of the items; coded 0 if answered no to all items
	Cursed, insulted, shouted at or humiliated you? Referred to your skin colour/gender/religion/tribe or health problems you have in a hurtful way? Stopped you from being with other children to make you feel bad or lonely? Tried to embarrass you because you were an orphan or without a parent? Embarrassed you because you were unable to buy things? Stole or broke or ruined your belongings? Threatened you with bad marks that you didn’t deserve? Accused you of witchcraft?	
	Physical violence	
	Hurt you or caused pain to you? Slapped you with a hand on your face or head as punishment? Slapped you with a hand on your arm or hand? Twisted your ear as punishment? Twisted your arm as punishment? Pulled your hair as punishment? Hit you by throwing an object at you? Hit you with a closed fist? Hit you with a stick? Caned you? Kicked you? Knocked you on the head as punishment? Made you dig, slash a field, or do other labour as punishment? Hit your fingers or hands with an object as punishment? Crushed your fingers or hands as punishment? Made you stand/kneel in a way that hurts to punish you? Made you stay outside for example in the heat or rain to punish you? Burnt you as punishment? Taken your food away from you as punishment? Forced you to do something that was dangerous? Choked you? Tied you up with a rope or belt at school? Tried to cut you purposefully with a sharp object? Severely beat you up?	
	Sexual violence	
	Teased you or made sexual comments about your breasts, genitals, buttocks or other body parts? Touched your body in a sexual way or in a way that made you uncomfortable? Showed you pictures, magazines, or movies of people or children doing sexual things? Made you take your clothes off when it was not for a medical reason? Opened or took their own clothes off in front of you when they should not have done so? Kiss you when you didn’t want to be kissed? Make you touch their genitals, breasts or buttocks when you didn’t want to? Touch your genitals, breasts or buttocks when you didn’t want them to? Give you money/things to do sexual things? Involve you in making sexual pictures or videos? Threaten or pressure you to have sex or do sexual things with them? Actually make you have sex with them by threatening or pressuring you, or by making you afraid of what they might do? Make you have sex with them by physically forcing you (have sex with you)?	
Violence peers (male and/or female student) past week	Emotional violence	Coded 1 if answered yes about past week experience to any of the items; coded 0 if answered no to all items
	Insulted you, or called you rude or hurtful names? Accused you of witchcraft?	
	Physical violence	
	Locked you out or made you stay outside? Not given you food? Twisted your arm or any other body part, slapped you, pushed you or thrown something at you? Punched you, kicked you, or hit you with a closed fist? Hit you with an object, such as a stick or a cane, or whipped you? Cut you with a sharp object or burnt you?	
	Sexual violence	
	Disturbed or bothered you by making sexual comments about you? Kissed you, when you did not want them to? Touched your genitals or breasts when you did not want them to, or in a way that made you uncomfortable? Threaten or pressure you to make you do something sexual with them? Make you have sex with them, because they threatened or pressured you? Had sex with you, by physically forcing you?	
Involvement in making up rules	Have you ever been involved in making up rules for how students should behave at your school?	0 = no
		1 = yes
Consideration of students’ views	In your school, are students’ views about how to improve the school taken seriously by adults who work at the school? Would you say all the time, most of the time, sometimes, or never?	0 = all the time
		1 = most of the time
		2 = sometimes
		3 = never
School connectedness	I feel that my teachers care about me; I feel safe in school; I feel like I belong at school; I like to spend time at school	each question:
		1 = all the time
		2 = most of the time
		3 = sometimes
		4 = never
		summary measure:
		0 = high
		1 = medium
		2 = low
Connectedness to peers	I feel close to students at my school	0 = all the time
		1 = most of the time
		2 = sometimes
		3 = never

#### Violence school staff past week and violence peers past week

Most questions about emotional, physical, and sexual violence were adapted from the ISPCAN Child Abuse Screening Tool Children’s Version Institutional Version (ICAST-CI) [26] and some questions from the WHO Multi-country Study on Women’s Health and Domestic Violence against Women [27]. Children were categorized as having experienced violence from school staff in the past week if they had experienced at least one out of 45 different violent behaviors in the past week. Children were also asked about 14 different violent behaviors from other students (peers) and coded as having experienced violence if they reported that they experienced at least one of the behaviors.

#### Involvement in making up rules and consideration of students’ views

Students were asked ‘Have you ever been involved in making up rules for how students should behave at your school?’. They were also asked to what extent their views about how to improve the school are taken seriously by adults who work at the school. The answers to these questions were used to assess student participation.

#### School connectedness

The questionnaire included four connectedness items that were similar to measures commonly used in adolescent health behavior surveys like the United States’ National Longitudinal Study of Adolescent Health (Add Health) [11]. To obtain a summary measure, the mean value of the four answers was calculated for each student and then cut-offs classifying students into three approximately equal groups were determined (high, medium and low school connectedness). Cronbach’s alpha for this scale was 0.62.

#### Connectedness to peers

Students answered the statement ‘I feel close to students at my school’ on a four-point-scale.

#### School size

The total number of students in Primary 5, 6, and 7 was taken as a proxy, because we had accurate class lists for these years from the Good Schools Study.

#### School location

Schools were classified as urban or rural by a member of Raising Voices staff with close knowledge of the district and study schools.

#### Mental health difficulties

The self-report version of the Strengths and Difficulties Questionnaire (SDQ) was used to measure mental health [28]. For each child, the Total Difficulties Score was calculated by summing the responses to 20 items covering emotional symptoms, conduct problems, hyperactivity-inattention and peer problems. The lowest possible value is 0 (good mental health), the highest is 40 (poor mental health). Cronbach’s alpha of the score of the sample in this paper was 0.69. As it has been suggested that approximately 20 % of a community sample can be categorized as ‘borderline/abnormal’ [29], a binary variable comparing children scoring above (‘children with mental health difficulties’) and below the 80^th^ percentile was created.

#### Confounding variables

*Age* and *sex* were *a priori* defined as confounders. To adjust for socio-economic status (SES), the variable indicating how many *meals* the child had eaten the previous day was taken as a proxy. *Disability* (trouble seeing, hearing, walking/with movement, with speech, fits, other) was regarded as a potential confounder as there is evidence that children with disabilities are at higher risk of experiencing violence [21, 30] and might be more likely to experience mental health problems compared to their non-disabled peers. Furthermore, the *school year attended* (Primary 5, 6, and 7) was considered. It was regarded as reasonable to control for experiences of *violence from relatives in the past week* and experiences of *violence from relatives more than 12 months ago* as studies have shown that experiences of violence from one type of perpetrator are associated with experiences of violence from other types of perpetrator [31]. For the assessment of *violence from relatives*, the same 14 different types of violence and the same coding (experienced at least one of the behaviors) as for *violence from peers* were used. Furthermore, experiences of *violence from school staff more than 12 months ago* and experiences of *violence from peers more than 12 months ago* were included to control for past experiences. Again, the same items and coding were used as for the assessment of experiences of *violence from school staff in the past week* and experiences of *violence from peers in the past week*.

### Data analysis

Multilevel logistic regression was applied to examine the associations between school-related factors and mental health [32, 33]. This analysis method accounts for the clustered nature of the data, i.e. that students (level 1 units) are nested within schools (level 2 units), which is necessary for obtaining correct regression estimates. Furthermore, the approach enables the assessment of the role of the context on individual health outcomes [34]. A random effect for school was added to each fitted model to allow the intercept to vary across schools (random intercept model). A complete-case analysis was conducted, i.e. students with missing or non-substantive values (‘don’t know’, ‘no response’, ‘not applicable’) in one or more variables of interest were excluded from the analysis.

First, a model with the outcome but no exposure and confounding variables was fitted to estimate the intra-class correlation (ICC) and to assess evidence for clustering based on the provided likelihood ratio test (LRT). In order to have guidance for the multivariable analysis, a conceptual framework was created (Fig. 1) [35].

MODEL 1 contained all variables shown in the framework except school connectedness and connectedness to peers, in order to exclude variables that we hypothesized might lie on the causal pathway between violence and mental health and participation and mental health, respectively. MODEL 2 comprised all variables. Partial mediation was regarded as having occurred if the effect of the exposure variables on the outcome had decreased by at least 10 % in MODEL 2 compared to MODEL 1.

To quantify the school-level variation (i.e. investigate the role of the context), median odds ratios (MORs) were calculated [34]. This measure conveys how much the odds of mental health difficulties of a student would (in median) increase if he/she moved to a school where the odds of mental health difficulties was higher. The MOR can be directly compared to the ORs of individual (e.g. age, sex) or area (e.g. urban/rural) variables. The MOR is always larger than or equal to one, with the value one indicating no difference in the probability for mental health difficulties between schools. Lastly, LRTs were used to investigate whether the effect of one or more exposure variables on the outcome differed between boys and girls. A p-value below 0.05 was considered as statistically significant. All analyses were conducted using STATA 12 [36].

## Results

### Descriptive statistics

The participation rate of those sampled was 77 %. The reason for non-participation was mainly absenteeism from school during the period of data collection (19 %). Hence, 3,706 students participated in the study of which 52.3 % were girls (Table 2). The majority of students (81.4 %) were between 11 and 14 years old. Over half of students experienced violence from school staff in the past week. The prevalence of violence from peers in the past week was 21.1 %. Almost half of students were involved in making up rules in the past and most students (62.6 %) perceived that student’s views are considered all the time or most of the time. The majority felt close to other students all the time or most of the time (78.7 %). With a median of 8 and a range from 0 to 30 points, the Total Difficulties Score was highly skewed to the right. The 20.7 % that were categorized as children with mental health difficulties had scores of 14 and higher. The total number of students in Primary 5, 6, and 7 (school size) in each school varied between 73 and 734 (median = 135.5). The majority of schools was located in a rural area (64.3 %).

**Table 2:** Student- and school-level characteristics

Student-level variables (Level 1)	*N* ^a^ (%)^b^ (*N* = 3,706)
*Socio*-*demographic characteristics*	
Age group, years	
7 to 10	159 (4.3)
11 to 14	3,011 (81.4)
15 to 18	531 (14.4)
Female sex	1,937 (52.3)
Meals eaten yesterday	
3+	1,743 (47.0)
2	1,443 (39.0)
1 or no meal	519 (14.0)
School year attended	
Primary 5	1,452 (39.2)
Primary 6	1,327 (35.8)
Primary 7	927 (25.0)
Disability	271 (7.3)
*Violence*	
Violence school staff past week	2,057 (55.5)
Violence school staff >12 months	3,097 (83.6)
Violence peers past week	782 (21.1)
Violence peers >12 months	999 (27.0)
Violence relatives past week	223 (6.0)
Violence relatives >12 months	763 (20.6)
*Participation in school life*	
Involvement in making up rules	1,704 (46.3)
Consideration of students’ views	
All the time	1,189 (32.9)
Most of the time	1,073 (29.7)
Sometimes	937 (25.9)
Never	414 (11.5)
*Connectedness*	
School connectedness	
High	859 (23.3)
Medium	1,524 (41.4)
Low	1,303 (35.4)
Connectedness to peers	
All the time	1,799 (48.6)
Most of the time	1,112 (30.1)
Sometimes	676 (18.3)
Never	112 (3.0)
*Mental health*	
SDQ categorical	
Normal	2,938 (79.3)
Borderline/abnormal	768 (20.7)
SDQ - Total Difficulties Score, median (range)	8 (0, 30)
School-level variables (Level 2)	*N* ^a^ (%)^b^ (*N* = 42)
School size, median (range)	135.5 (73, 734)
School location	
Rural	27 (64.3)
Urban	15 (35.7)

^a^If not indicated as median; totals vary due to missing data and non-substantive responses (‘don’t know’, ‘no response’, ‘not applicable’): age: 5, meals eaten yesterday: 1, involvement in making up rules: 28, consideration of students' views: 93, school connectedness: 20, connectedness to peers: 7

^b^If not indicated as range; % calculated on valid responses

### Associations between school-related factors and children’s mental health

Data were incomplete for only a small proportion of students (3.8 %, *N* = 141), hence 3,565 students were included in the analysis. The distributions of key variables like age, sex, experiences of violence from school staff and peers in the past week, school connectedness and connectedness to peers were similar for students with incomplete records compared to those with complete records.

The ICC was estimated to be 4 %, indicating that 4 % of the variance in the propensity for mental health difficulties is attributable to differences between schools. The LRT yielded a value of *p* < 0.001, providing strong evidence for clustering and therefore justifying a multilevel model.

#### MODEL 1: association of violence, participation in school life, and school characteristics with mental health

Experiences of violence from school staff and peers in the past week were both strongly associated with mental health difficulties (OR = 1.58, 95 % CI 1.31 to 1.90 and OR = 1.81, 1.47 to 2.23, respectively) (Table 3). Past involvement in making up rules for how students should behave at school was associated with a 1.40 times (1.18 to 1.67) higher odds of mental health difficulties. Compared to students who felt that adults working at the school always take students’ views about how to improve the school seriously, those who felt that this was never the case had a higher odds of mental health difficulties (OR = 1.33, 0.99 to 1.77). However, overall the statistical evidence for this association was weak (*p* = 0.105). School size was not associated with mental health difficulties (OR = 1.01, 0.89 to 1.15), however, attending a school in an urban area was associated with an increased odds of mental health difficulties (OR = 1.44, 1.05 to 1.98).

**Table 3:** Factors associated with mental health difficulties – multivariable analysis

	MODEL 1^a^ (*N* = 3,565)		MODEL 2^b^ (*N* = 3,565)	
	OR (95 % CI)	*p* value^c^	OR (95 % CI)	*p* value^c^
Student-level variables (Level 1)				
*Socio*-*demographic characteristics*				
Age (per 1 year increase)	1.09 (1.02–1.17)	0.014	1.08 (1.01–1.16)	0.024
Female sex	1.20 (1.01–1.44)	0.043	1.14 (0.95–1.37)	0.159
Meals eaten yesterday				
3+	1		1	
2	1.44 (1.19–1.74)		1.42 (1.17–1.72)	
1 or no meal	1.98 (1.55–2.53)	<0.001	1.91 (1.49–2.45)	<0.001
School year attended				
Primary 5	1		1	
Primary 6	0.99 (0.81–1.22)		1.00 (0.82–1.23)	
Primary 7	0.59 (0.45–0.77)	<0.001	0.60 (0.46–0.78)	<0.001
Disability	1.53 (1.14–2.06)	0.005	1.49 (1.11–2.01)	0.010
*Violence*				
Violence school staff past week	1.58 (1.31–1.90)	<0.001	1.56 (1.29–1.88)	<0.001
Violence school staff >12 months	1.22 (0.94–1.60)	0.136	1.20 (0.92–1.57)	0.179
Violence peers past week	1.81 (1.47–2.23)	<0.001	1.80 (1.46–2.21)	<0.001
Violence peers >12 months	1.09 (0.89–1.34)	0.391	1.09 (0.89–1.34)	0.397
Violence relatives past week	1.70 (1.20–2.41)	0.003	1.67 (1.17–2.37)	0.005
Violence relatives >12 months	0.97 (0.77–1.22)	0.783	0.94 (0.74–1.19)	0.624
*Participation in school life*				
Involvement in making up rules	1.40 (1.18–1.67)	<0.001	1.43 (1.20–1.71)	<0.001
Consideration of students’ views				
All the time	1		1	
Most of the time	1.27 (1.02–1.57)		1.14 (0.91–1.42)	
Sometimes	1.12 (0.89–1.41)		0.99 (0.78–1.26)	
Never	1.33 (0.99–1.77)	0.105	1.12 (0.83–1.51)	0.554
*Connectedness*				
School connectedness				
High			1	
Medium			1.15 (0.90–1.46)	
Low			1.43 (1.11–1.83)	0.011
Connectedness to peers				
All the time			1	
Most of the time			1.12 (0.91–1.39)	
Sometimes			1.94 (1.55–2.44)	
Never			1.86 (1.18–2.93)	<0.001
School-level variables (Level 2)				
School size (per 100 students increase)	1.01 (0.89–1.15)	0.862	0.99 (0.88–1.12)	0.908
School location				
Rural	1		1	
Urban	1.44 (1.05–1.98)	0.026	1.37 (1.01–1.88)	0.051
Random effects				
Between-school variance (SE)	0.1139 (0.0431)		0.1102 (0.0425)	
Median odds ratio	1.38		1.37	

^a^Variables included: age, sex, meals eaten yesterday, school year attended, disability, violence school staff past week, violence school staff >12 months, violence peers past week, violence peers >12 months, violence relatives past week, violence relatives >12 months, involvement in making up rules, consideration of students’ views, school size, school location

^b^Variables included: all variables in MODEL 1 plus school connectedness, connectedness to peers

^c^Likelihood ratio test

#### MODEL 2: association of connectedness factors with mental health

There was good evidence for an association between school connectedness and mental health difficulties (Table 3). The OR comparing those with a low school connectedness to those with a high school connectedness was 1.43 (1.11 to 1.83). Never feeling close to other students at school was also associated with mental health difficulties (OR = 1.86, 1.18 to 2.93).

### Mediation of the effects of violence and participation on mental health by connectedness

We did not find any evidence that school and peer connectedness mediated the association between violence and mental health, or between involvement in making up rules and mental health. Only the OR’s for consideration of students’ views were between 10 and 15 % lower in MODEL 2 compared to MODEL 1; indicating that connectedness partially mediated the association between consideration of views and mental health.

### School-level variation

The MORs were 1.38 and 1.37 in MODEL 1 and MODEL 2, respectively (i.e. if a student moved to another school with a higher probability of mental health difficulties, the student’s odds of mental health difficulties would (in median) increase by around 1.4 times).

### Effect modification by sex

Our tests did not show any statistical evidence of a difference in effects of any of the individual-level exposure variables on mental health between boys and girls. The LRTs comparing models with an interaction term between each exposure variable and sex with a model without this term yielded p-values of 0.182 (different effect of ‘violence from peers in the past week’ on mental health for boys and girls in MODEL 1) and larger, indicating that the less complex model fitted better.

## Discussion

The results of our analyses supported our hypothesis that both experiences of violence from school staff and peers at school in the past week and low levels of school and peer connectedness would be associated with increased levels of mental health difficulties in students. We found that the relationship between experiences of violence from school staff and peers and mental health difficulties is not mediated by connectedness factors, and that both violence and connectedness factors contribute independently to mental health difficulties. Furthermore, we found no evidence that associations differed by sex. As expected, our results show that even after accounting for individual levels of violence experience and socio-demographic factors, and connectedness, students in urban schools were more likely to report poor mental health versus students in rural schools.

Our findings support existing studies from both developed and developing countries, which reported that being bullied is associated with internalizing problems, loneliness, sadness, psychosomatic symptoms and/or suicidal ideation [15–20]. Bullying is a specific type of school violence [14] which comprises repeated physical, verbal or psychological attacks or intimidation and is characterized by an imbalance of power, i.e. a more powerful child or group of children oppresses a less powerful one [37]. The Good Schools Study did not aim to assess bullying as such. However, some of the physical and emotional violence experiences we asked about are often described as bullying behavior (e.g. name calling, kicking/hitting).

Our findings are also consistent with other cross-sectional [11] and longitudinal [8–10] studies that found that a good school connectedness or socio-educational environment was concurrently associated with or predictive of fewer depressive symptoms and anxiety in later years.

To our knowledge, this is the first study to examine whether school connectedness mediates the relationship between violence experiences and poor mental health, and we did not find evidence in support of this pathway. Exposure to the physical violence we measured in this survey is normative in the Ugandan school context, which may also mean that experience of physical violence is less stigmatising and therefore less likely to affect social relationships and connections with peers and school in such a way that mental health is adversely impacted.

In contrast with other studies, our findings did not support the hypothesis of an association between low participation in school life and mental health difficulties. The unexpected finding that involvement in making up rules is associated with mental health difficulties was not replicated in any other study. The Irish Health Behavior in School-aged Children survey showed that school participation (organizing school events, encouragement of expressing views in class, making the school rules) was associated with higher self-rated health and happiness [12]. Conversely, a New Zealand study found no evidence for an association between student participation and either depression symptoms or suicide behaviors [13]. The association between involvement in making up rules and mental health difficulties found in our study could potentially be explained by reverse causality, i.e. students with mental health difficulties might behave inappropriately and be called to disciplinary meetings or otherwise discuss plans to change their behaviour, and then may report this as being involved in making up rules. However, further investigation would be needed to confirm this.

Studies conducted in high-income countries have found no evidence for an association between school size and mental health outcomes [8, 13]. Our study using data from a low-income country also showed no association between school size and mental health.

Other research on the effects of urban versus rural residence on adult mental health has also found an increased risk of mental disorders in urban areas [38], with some evidence that this is due to increased stress experienced by urbanites [39]. Further research is needed to understand what affects students’ mental health in this context. We note that in our sample, several of the urban schools were highly academically oriented and selected by parents on the basis of having excellent exam results. This may indicate an environment where students are under pressure to achieve good exam results, which in turn may compromise mental health.

In addition to overall worse mental health in urban schools, our findings also indicate unexplained residual variation in mental health difficulties between schools (after accounting for all factors in the model). Further research is clearly needed to understand what is putting students in some schools at higher risk.

### Limitations

This study is one of very few rigorously conducted studies investigating the relationship between various factors related to school and mental health of students in a developing country, however, it is cross-sectional, and results should not be interpreted as causal.

While the proportion of sampled children who completed the survey within schools was quite high (77 %), selection bias cannot be completely ruled out. The main reason for non-participation was being absent during the data collection period. This may in turn be associated with working outside of school. Previously published results from this study indicated that the odds of experiencing different forms of violence was higher for those working one hour or more a day outside of school compared to those working less than one hour [21, 22]. Absenteeism may also be associated with poorer (mental) health status, which would mean our analyses may have underestimated the magnitude of the association between violence and mental health difficulties.

Questions about violence were taken from the ICAST-CI which has been widely tested and used internationally [26]. Students were repeatedly assured that teachers and staff members would not find out what they had answered to prevent underreporting of violence experiences. Nevertheless, underreporting might have occurred because of the shame and stigma which can be associated with some forms of abuse (e.g. sexual abuse) [27], or students may have had difficulty recalling some exposures. The main focus of the Good Schools Study was examining violence from school staff, hence more questions on this form of violence were included than on violence from other perpetrators. It is therefore possible that we failed to categorize some students as having experienced violence from others besides school staff because of the less detailed inquiry about violence experiences. For the assessment of mental health, the SDQ, a well-established and widely used instrument, was used. It should be noted that although having high levels of mental health difficulties on a screening tool such as the SDQ has a strong relationship with subthreshold and full-blown psychiatric disorders, a high SDQ score should not be interpreted as a psychiatric diagnosis [28, 29].

Lastly, the study was conducted only in one district of Uganda. However, the population in this district is demographically similar to the rest of the country and has both rural and urban areas [23]. Therefore, the results from this study are likely to be generalizable to other primary school students in Uganda.

### Implications

Our results suggest that interventions for young people attending school should address their experiences of violence from school staff and from peers to improve mental health. Programs targeting bullying and other forms of violence in schools which have been developed in high - income countries and been proven to be effective should urgently be explored for their efficacy in low-income settings [37].

School connectedness is protective against student risk-taking behavior like substance use and positively impacts on educational outcomes [7, 40]. Fostering school connectedness is therefore a good strategy for strengthening mental health of students as well as for promoting other aspects of students’ health and academic achievements. Strategies for improving school connectedness have been integrated in existing intervention programs [40], and recommendations for increasing school connectedness have been developed by the Centers for Disease Control and Prevention that can serve as a practical guide for teachers [7].

## Conclusions

Recent experiences of violence from school staff and peers at school, as well as poor school connectedness and poor connectedness to other students, are associated with mental health difficulties in Ugandan primary school students, but the effect of violence is not mediated through poor connectedness. Students in urban schools are at higher risk for poor mental health, even after individual exposures to violence and connectedness are accounted for. Further research is needed to understand the elevated risk for urban students, and interventions to improve students’ mental health must address violence.

## Abbreviations

ICAST-CI, ISPCAN Child Abuse Screening Tool Children’s Version Institutional Version; SDQ, Strengths and Difficulties Questionnaire; WHO, World Health Organization
